# Let's get active: The use of technology‐enhanced audience interaction to promote active learning

**DOI:** 10.1002/aet2.10950

**Published:** 2024-05-19

**Authors:** Simanjit K. Mand, Stephen J. Cico, Mary R. C. Haas, Nicole E. Schnabel, Benjamin H. Schnapp

**Affiliations:** ^1^ BerbeeWalsh Department of Emergency Medicine University of Wisconsin School of Medicine and Public Health Madison Wisconsin USA; ^2^ Department of Clinical Sciences University of Central Florida College of Medicine Orlando Florida USA; ^3^ Department of Emergency Medicine University of Michigan Medical School Ann Arbor Michigan USA

## INTRODUCTION

Active learning, the process of engaging learners to partake in their education through participation and discussion, has gained significant traction in medical education over the past decade.^1^ Active learning methods enhance audience attentiveness and overall educational enjoyment.^2^, ^3^, ^4^ Recent literature also highlights enhanced knowledge acquisition and retention with active learning approaches compared to passive learning methods, indicating both immediate and potential long‐term benefits.^2^, ^3^, ^5^, ^6^, ^7^, ^8^, ^9^, ^10^

Active learning, however, has potential drawbacks. Within large group settings, it can inadvertently lead to learners feeling anxious, ashamed, or inadequate compared to their peers if it exposes knowledge gaps. This can hinder their ability to engage fully in the learning process.^11^, ^12^ It remains necessary to ensure learners are provided with psychological safety to concentrate solely on the learning task at hand without the risk of feeling self‐conscious among their peers.^13^

Technology‐enhanced audience interaction offers the advantage of promoting active learning while still ensuring psychological safety for learners. These platforms enable participation with the option of anonymity, addressing learner concerns about potential negative exposure to knowledge deficits and creating a supportive learning environment by encouraging participation by all.^4^ The versatility and diversity of options for engagement can allow for easy integration into a variety of existing educational resources. While technology‐enhanced audience interaction can be used in a variety of educational environments and situations, here we explore key considerations for use in large group settings.

## CONSIDERATIONS WHEN USING TECHNOLOGY‐ENHANCED AUDIENCE INTERACTION

Technology‐enhanced audience interaction can be accomplished using a variety of different software programs; factors related to the presentation content, targeted learners, software characteristics, and learning environment may influence the optimal software choice for a given learning activity. Software selection will first depend on the objectives of the presentation and intended level of learner participation. Presenters may wish to use certain software to gamify content (e.g., multiple‐choice questions, polls) to assess learner recall and retention of subject matter while encouraging friendly competition to maintain attention. Others may wish to use software to perform a real‐time needs assessment of their audience to tailor education delivery; presenters can ask learners questions and, based on accuracy of responses, focus subsequent teaching material on topics which the learners have not yet mastered. Even further, a presenter may want to choose software that offers whiteboards to facilitate discussion‐based sessions and collaborative knowledge building in environments such as a flipped‐classroom.

While certain learning environments may benefit from presenters being able to clearly identify participants, such as situations needing individual assessment and feedback, others may offer anonymity while still allowing audience members to complete self‐assessments. In presentations involving large groups, such as national conferences or Grand Rounds presentations, or settings with less familiarity among the presenter and learner audience, anonymity can allow for greater audience participation. Furthermore, in sessions meant to engage students or trainees, or presentations covering sensitive subject matter, anonymity may enhance psychological safety for both audience responses and questions to promote a safe learning environment. Anonymous formats may also provide a medium for presenters to receive honest learner input or feedback.

Once the intent of software use has been established, software‐specific factors such as integrability, ease of use, and data analysis capability are several characteristics for a presenter to consider. The amount of time required to incorporate the software into their learning plan is one primary factor that may impact choice. Several software programs offer premade graphics and templates that enhance efficiency (e.g., Mentimeter, Padlet, Slido), while others have fewer templates that may appeal to those who wish to exercise their creativity or use a more individualized format. Software programs also vary in their interface for participants; some software options utilize a unique code with which participants can easily join from their personal device, while others require the download of an application or a tedious sign‐up and log‐in process. Lastly, the option to obtain summative reports from the presentation for further use may also impact which software program is selected (see Table 1 for a comparison of features for various software platforms).

**TABLE 1:** Audience interaction software programs.

Audience interaction software	Available features	Ideal educational use	Presentation integration^a^	Free account features	Log‐in process
Poll Everywhere https://www.polleverywhere.com 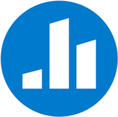	*Question types*: Multiple choice Word cloud Open‐ended Survey *Assessment*: Analytics	To perform comprehensive assessment during/end of a lecture To promote gamification of content	Yes	25 participants Unlimited questions Event analytics	*Presenter*: Sign up *Participant*: URL from presentation
Slido https://www.slido.com 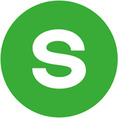	*Question types*: Multiple choice Word cloud Open‐ended Poll Quiz *Assessment*: Analytics	To perform comprehensive assessment during/end of a lecture	Yes	100 participants Unlimited questions Three polls/event One quiz/event Event analytics	*Presenter*: Sign up *Participant*: Event code from presentation
Plickers https://www.plickers.com 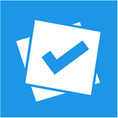	*Question types*: Multiple choice *Assessment*: Analytics	To perform comprehensive assessment during/end of a lecture	No; operates on Plickers application after download	63 participants Unlimited sets of five questions Event analytics	*Presenter*: Sign up, download app, add participants, assign card numbers, scan Plickers card answers *Participant*: Hold up card
Kahoot! https://kahoot.com 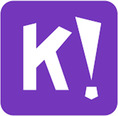	*Question types*: Multiple choice Word cloud Open‐ended Poll True/false *Assessment*: Analytics	To perform comprehensive assessment during/end of a lecture To promote gamification of content	Yes; download add‐on for primary presentation application	40 participants	*Presenter*: Sign up *Participant*: Event code from presentation
Mentimeter https://www.mentimeter.com 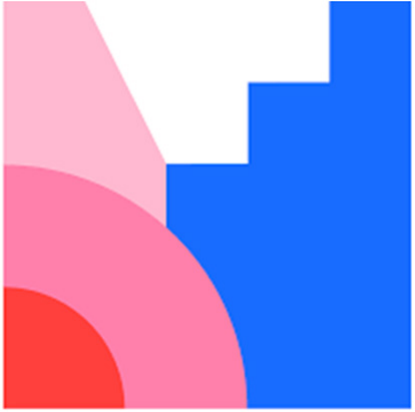	*Question types*: Word cloud Open‐ended Poll Quiz Survey	To perform comprehensive assessment during/end of a lecture To create a participant survey for your presentation To use a new software program for your presentation	Yes; download add‐on for primary presentation application Option to create presentation content in program	50 participants/month Limited question types	*Presenter*: Sign up *Participant*: Enter code from presentation
Quizizz https://quizizz.com 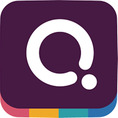	*Question types*: Multiple choice Word cloud Open‐ended Poll Flash cards *Assessment*: Analytics	To perform comprehensive assessment during/end of a lecture To create asynchronous, interactive learning plan with instant self‐assessment	No, create presentation content in program	50 participants All question types Event analytics Limited free trial (30 days)^a^	*Presenter*: Sign up *Participant*: Enter code from presentation
Jamboard https://jamboard.google.com 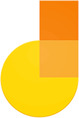 no longer available starting Oct 2024^a^	Virtual whiteboard	To use in situations desiring group‐think such as brainstorming, diagramming, and mapping	No; option to use with virtual platforms (i.e., Google Slides)	Entire application	*Presenter*: Sign in with Google email *Participant*: Sign in and join document sent by presenter
Miro http://miro.com 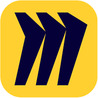	Virtual whiteboard	To use in situations desiring group‐think such as brainstorming, diagramming, and mapping	No; option to use with virtual platforms (i.e., Zoom)	3 editable boards 2500+ templates	*Presenter*: Sign up *Participant*: Sign up and join document sent by presenter
Padlet https://padlet.com 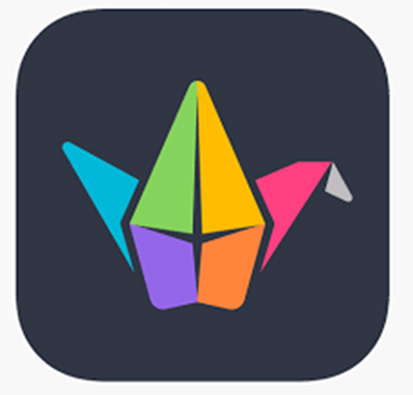	Virtual whiteboard	To use in situations desiring group‐think such as brainstorming, diagramming, and mapping To elevate presentations rich in visual content—media, videos To promote material on blog or website	No	Three editable boards 20 MB/upload	*Presenter*: Sign up *Participant*: Sign up and join document sent by presenter
Zoom https://zoom.us 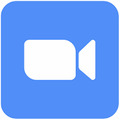	*Question types*: Polls Virtual whiteboard	To perform comprehensive assessment on virtual platform	No	Meetings (up to 40 min/meeting) 100 attendees/meeting	*Presenter*: Sign up *Participant*: Click link for meeting
FigJam https://www.figma.com 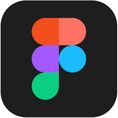	Virtual whiteboard	To use in situations desiring group‐think such as brainstorming, diagramming, and mapping To design basic graphics for presentation or pamphlet use	No	Unlimited collaborators Mobile application available Limited free trial (30 days)^a^	*Presenter*: Sign up *Participant*: Sign up and join document sent by presenter
LucidSpark https://lucid.app 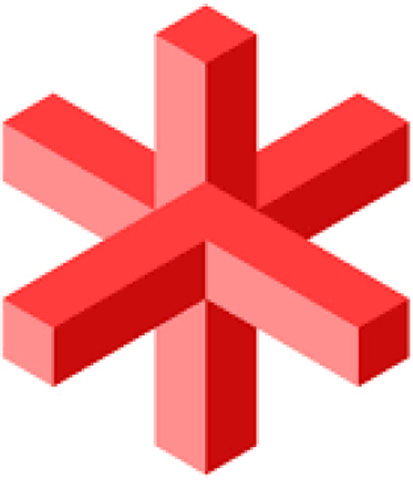	Virtual whiteboard	To use in situations desiring group‐think such as brainstorming, diagramming, and mapping To use a new software program for your presentation	Yes; download add‐on for primary presentation application Also option to use with virtual platforms (i.e. Google Slides) and create presentation content in‐program	Three editable boards 100 templates	*Presenter*: Sign up *Participant*: Sign up and join document sent by presenter

^a^ Presentation integration addresses use with the most common applications of PowerPoint and Keynote.

Presenters must now also consider if the presentation will take an in‐person, virtual, or hybrid format. Most software programs were developed to create an interactive and engaging in‐person experience, but several (e.g., Slido and PollEverywhere) are specifically marketed for their ability to incorporate easily into common virtual conferencing software programs. Other applications, such as Zoom, offer the ability to do audience polling and collaboration within the software program itself.

## POTENTIAL DOWNSIDES

Technology‐enhanced audience interaction can present challenges. First, technology can prove to be a distraction to learning. Learners will often send text messages, make calls, or do other activities on mobile devices during the time they are supposed to be learning.^14^ Second, there is always a risk for technology failure. This is particularly salient in large group or high‐stakes settings, such as presentations at national conferences or Grand Rounds. In unfamiliar locations, Wi‐Fi connections can be slow (e.g., public connections) and inconsistent, and sometimes certain types of domains are blocked or inaccessible.^15^ When at all possible, testing of technology should be completed before the presentation. Lastly, presenters must consider not only the extra time that can be required to reformat entire presentations, courses, or curricula to accommodate technology‐enhanced audience interaction, which may be in short supply in a busy academic practice, but also the amount of time required for audience members to log into or access the software platform during the learning activity.

An additional consideration for incorporating technology‐enhanced audience interaction is the cost for a subscription. Though most of the resources listed in Table 1 have free tiers, often those “free” or “trial” accounts come with limitations in use (see Table 1, Free Account Features column). These can include limiting the number of responses or participants, limits to the number of questions that can be asked, or limitations on the types or ways of presenting data. Some institutions and departments may have subscriptions to one or more platforms, but others do not. It can be a lengthy process to get approval to access these software programs and obtain subscriptions or available funds may be unable to be used to purchase licenses.^16^

Lastly, technology is ever‐changing; upgraded features become available for existing software platforms in addition to novel applications released on a seemingly daily basis. There is also risk for the migration or loss of familiar resources; Jamboard, currently available through Google, will no longer be accessible after October 2024. This endless evolvement of resources requires presenters to maintain familiarity with existing software platforms and have a pulse on up‐and‐coming resources that may better suit their needs. Because of the potential drawbacks of technology‐enhanced audience interaction, it is always worth considering whether a technological solution is the best one to create a safe, effective active learning environment for your learning activity or whether low or no technology solutions may be equally or more effective.

## CASE EXAMPLES

Case 1 You are revamping a session on airway management and want to gather from the audience what they already know about the topic. Previously, this had been done via raising hands, but typically only a few learners were willing to share. This year, you create a Jamboard so that all learners can simultaneously collaborate on a mindmap of how they conceptualize the various aspects of airway management.

Case 2 You are running an in‐training examination review session and are frustrated with it. To create more engagement, you initially created a PollEverywhere to allow learners to test themselves on the content but have found that the pace is frustratingly slow for all as you wait for everyone to respond. After considering other options, you decide to switch to Kahoot!, which successfully adds some friendly competition and allows covering more content due to the improved pace.

Case 3 Your virtual presentation on how to read radiology images feels unexciting—you're talking to a bunch of black rectangles, and it's difficult to get any feedback about whether your content is being understood or not. To create more opportunities for engagement, you enable Zoom's polling and stamps features for your talk and intersperse questions for the audience throughout, allowing residents to demonstrate that they can identify abnormal findings and allowing you to adjust your teaching in real time to highlight content they are struggling with.

## CONCLUSIONS

Technology‐enhanced audience interaction can offer a way to create additional active learning opportunities for your presentation while protecting the psychological safety of your learners. While technology offers the promise of exciting and novel ways for creating this environment, it is not the only way to do so and can have potential downsides. The presenter should carefully consider whether an audience response software is a good fit for the targeted audience, content, and environment.

## AUTHOR CONTRIBUTIONS

All authors contributed to the study concept and design, drafting of the manuscript, critical and revision of the manuscript for important intellectual content.

## CONFLICT OF INTEREST STATEMENT

The authors declare no conflicts of interest.
